# A critical need for the concept of matrescence in perinatal psychiatry

**DOI:** 10.3389/fpsyt.2024.1364845

**Published:** 2024-06-10

**Authors:** Aurelie M. Athan

**Affiliations:** Teachers College, Columbia University, New York City, NY, United States

**Keywords:** matrescence, transition to motherhood, maternal mental health, perinatal psychiatry, Perinatal Mood and Anxiety Disorders (PMADs), reproductive health education

## Abstract

The concept of matrescence, akin to adolescence but for mothers, has gained increasing attention in perinatal psychiatry, marking a paradigm shift towards understanding the holistic development of mothers. Matrescence encompasses the myriad psychological, social, cultural, and existential changes which occur as women transition into motherhood. Despite advances in maternal mental health, a bias towards pathologizing maternal experiences persists in research and practice. This commentary advocates for the integration of matrescence into perinatal psychiatry, drawing from the work of Dana Raphael and contemporary scholarship. Matrescence offers a strengths-based framework that acknowledges both the challenges and opportunities of motherhood, emphasizing the normative aspects of a mother’s self-development. By adopting matrescence terminology and nosology, clinicians and researchers can enhance traditional psychiatric classifications. Additionally, matrescence underscores the importance of considering ecological systems and historical factors in maternal well-being, highlighting the need for comprehensive and compassionate healthcare services. Embracing matrescence as a fundamental concept in perinatal psychiatry holds promise for improving maternal mental health outcomes and promoting the flourishing of mothers worldwide.

## Introduction

Since the inception of psychiatry at the end of the 19^th^ century, its various theoretical orientations have shifted in prominence but rarely has the mother as a figure of interest for the well-being of individuals and the broader societal fabric lost her primacy. Mothers have been identified as primary agents in the developmental trajectories of their offspring and as sources of negative impact when in distress. Regrettably, this scholarly fascination has historically led to the stigmatization and medicalization of maternal experience without the same attention paid to their well-being. Over the past six decades, with the establishment of entities such as the International Society for Psychosomatic Obstetrics and Gynecology, the Marcé Society, and Postpartum Support International, the field of perinatal psychiatry has made significant contributions to recognizing the pivotal role of reproductive transitions in women’s health, facilitated by a global consortium of researchers, clinicians, educators, and advocates (1–4). This unfolded in parallel with social justice activism that ultimately led to legislative actions mandating the universal screening of new mothers for depression and their active participation in clinical research (5–8). By the turn of the 21st century, the field of maternal mental health had grown beyond its myopic focus on child outcomes to encompass a wider classification of diagnoses (e.g., perinatal mood and anxiety disorders or PMADs), clinical windows (e.g., prenatal onset), pathophysiologies (e.g., traumatic childbirth), interventions (e.g., zuranolone), and trainings (e.g., specialized curricula)— this time to alleviate the distress of the mothers themselves (9–11). Epidemiological data revealed that vulnerability in motherhood was ubiquitous with estimates between 15% to 21% of the general population experiencing depression, and up to 80% of mothers reporting distress of some kind (12). This paradigmatic shift from viewing the mother merely as a functional object to recognizing her as a psychological *subject* marked a belated but critical turn.

Despite these notable advancements, it is imperative to acknowledge that a deeply rooted biomedical “psychiatry bias” in maternal mental health research and practice still remains, inviting critical reflection (13). Ongoing theoretical innovation is necessary to advance bold ideas that can evolve outdated explanatory frameworks and guide scientific exploration now in the direction of building *resilience*. In 2018, the World Health Organization (WHO) underscored the mental health of mothers as a fundamental component of their overall health, defining maternal well-being not merely as a reductionistic absence of mental illness, but as the capacity for *flourishing* (14). There exists an urgent need for a strengths-based framework that studies the normative aspects of a mother’s self-development and acknowledges both the positive and negative outcomes with equal consideration. Such an expanded viewpoint provides a more comprehensive appreciation of mothers as complete individuals capable of thriving if given the optimal environmental conditions. Using a term known as *matrescence* from the archives of women’s health literature and, notably, from a different discipline, is a timely intervention (15). The process of becoming a mother, or *matrescence*, was first introduced in the 1970s by Dana Raphael, Ph.D., a female medical anthropologist who studied birth and breastfeeding and also coined the word ‘doula’. It was largely neglected until half a century later when the author revived it to complement diagnostic views in maternal mental health and expanded its definition further (15). Matrescence must once again evolve beyond its original conceptualization in order to offer a new lineage of thinking for the field of perinatal psychiatry. The larger promise of matrescence may be in its ability to reshape entrenched societal beliefs and, by extension, how health systems nurture maternal welfare by providing a more comprehensive and compassionate approach to their care.

## Matrescence terminology and nosology

In her pioneering yet overlooked work, *Becoming a Mother, A New/Old Rite de Passage in Reproduction, Power, and Change* (1975), Raphael stated,

*“The critical transition period which has been missed is matrescence, the time of mother-becoming … Giving birth does not automatically make a mother out of a woman … The amount of time it takes to become a mother needs study” (16, p.65-70).*

Raphael’s words serve as a call to inquire further into the nature of maternal psychological maturation and to integrate these insights into modern psychiatric theory and practice. The delayed explication of matrescence may be due, in part, to the lack of language available to describe new motherhood as a “life crisis” without cloaking it entirely in negative terminology. Like the term adolescence from the Latin word “adolescere”, meaning “to emerge, grow, or mature”, it may share similar defining features of developmental growth or regression true of any life transition. The more common terms of postpartum or perinatal are borrowed from the obstetric establishment and as such form a natural pairing with words of infirmity (e.g., postpartum depression). It illuminates how over time the study of maternal development has been saddled with a conceptual basis that has shaped it into a ‘curative discipline’ based on the medical model of disease, and to which some argue it largely owes its transformation into the subfield of perinatal psychiatry (17). Establishing its own unique nosology instead may help matrescence gain similar recognition and relevance. A standardized framework would also enable systematic investigation by others, enhance the quality and comparability of studies, and could play a vital role in education and awareness efforts.

**Figure 1:**
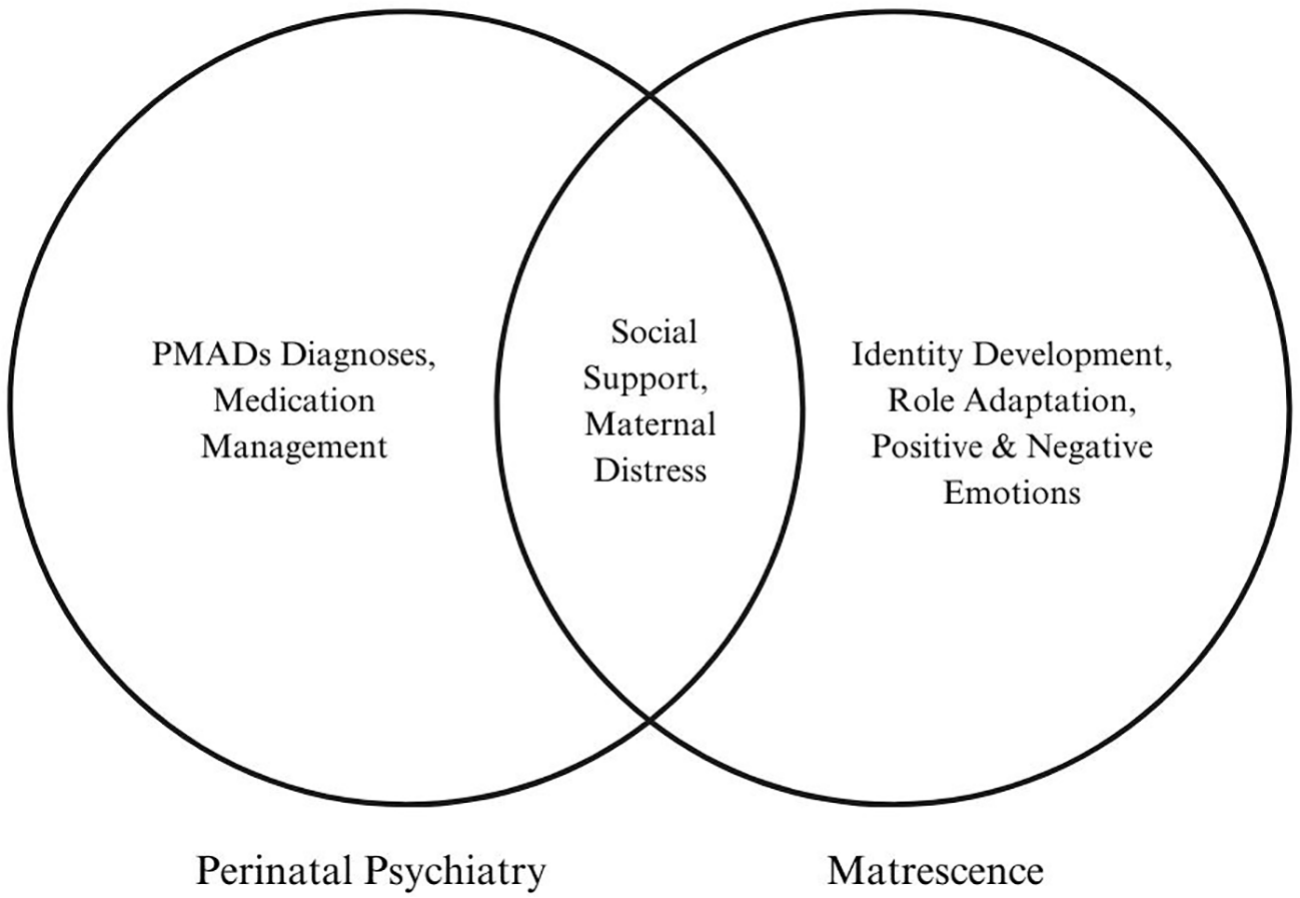
Clinical versus developmental perspectives.

The act of transcending disciplinary boundaries to adapt concepts from other fields is a key strategy for theoretical innovation. This approach not only enriches our understanding but also broadens our interpretative frameworks for the better (see *reproductive identity*) (18, 19). It is also an opportune moment as the classification of PMADs is currently being subjected to greater scrutinty (1). While more studies aim to distinguish conditions like postpartum depression from nonpuerperal major depression, comparisons to non-pathological human development remain curiously sparse. This discrepancy highlights a fundamental lack of understanding about what is *normative* during this life stage. More attention is needed as to whether general maternal distress is a distinct affective state, a subclinical presentation, or part of a normal continuum across the spectrum of illnesses from “baby blues” to psychosis (20). The utility of a developmental orientation such as matrescence is in helping to discern between normal and abnormal reactions to common experiences like fatigue or mood changes when interpreting the concerns of new mothers. For example, the term “perinatal distress” often serves as a vague catch-all that may incorrectly suggest impending illness (21). Recognizing it instead as a universal phenomenon of psychological disorientation to a major life change could clarify its meaning and reduce its potential overuse. In such an acute time of flux, distinguishing between psychiatric disorders and developmentally appropriate reactions can become challenging and potentially obscure the theoretical and practical boundaries between them (see **Figure 1**).

This conceptual “fuzziness” also extends into the history of nonspecific constructs often used to describe maternal psychology such as “transition to motherhood”, “becoming a mother”, “maternal role attainment”, or “maternal identity formation” (22). Using agreed-upon, succinct and clear terms such as Raphael’s *matrescence* and *matrescents* may reduce confusion, improve communication, and assist keyword searches when exploring these diverse bodies of literature. In terms of its timing and scope, precocious puberty and assisted reproductive technologies have widened the reproductive window and made it possible to become a mother even younger and older than previous generations. The exact length of matrescence is therefore individual, recurs with each child, and may last a lifetime with no clear endpoint. Integrating additional terminology from the field of developmental psychology could further improve cohesiveness and distinguish the various ages and stages of motherhood, similar to what is noted for young adulthood: *emerging motherhood* (infants to school-aged offspring), *middle motherhood* (adolescent offspring), and *late motherhood* (adult offspring). Notably, most research on maternal psychology predominantly centers around the early years of motherhood, leaving a gap in our understanding of late motherhood or the dynamics of parenting during menopause, especially considering the rising trend of delayed childbearing. This lack of comprehensive exploration suggests potential areas for future research to delve into the various phases of motherhood. For example, borrowing insights from the literature on grief could shed light on topics such as mourning the loss of one's former self or the emotional impact of the empty nest syndrome.

Raphael (16) gave us another clue that motherhood was a *holistic* experience with multidimensional significance: “The matrescent rite de passage can be examined as a biological fact, as a cultural event … The physiological stage of matrescence begins at the moment a female delivers a live infant. But, human beings are never limited by biological fact!” (16, pg. 66). Adolescence is widely recognized as a significant developmental period between childhood and adulthood that shares these equivalent all-encompassing changes. Although the comparison to adolescence is imperfect, theories from this field can provide valuable guidance in understanding the unique and distinct aspects of matrescence. Still, many questions remain unanswered. Is it a discrete life transition like adolescence or a complex life-long experience? If acute, what are the anticipated developmental tasks, and how does it then shape later adult development across the lifespan of a mother (and perhaps even into grandmothering)? What has been evidenced to date is that people who experience preconception, pregnancy, birth, surrogacy, adoption, and early parenting, undergo alterations not only in the few domains Raphael initially proposed but across *all* of the domains of human existence. With this in mind, the updated definition of matrescence for the 21st century offered here, as a first step in this longer inquiry process, is: a lifespan, developmental transformation that is biological, neurological, psychological, social, cultural, economic, political, moral, ecological, existential, and spiritual in nature. Furthermore, creating a comprehensive nosology for matrescence should also involve classifying both the developmental challenges *and* opportunities possible within each of these domains (see **Box 1**).

## Positive perspectives matter to patients

The exploration of the *positive psychology of motherhood* represents an essential aspect of matrescence that warrants further investigation into the opportunities for personal growth available to mothers. Perinatal patients often challenge clinical interpretations that solely pathologize their maternal experiences. Intolerance for this one-sidedness is gaining momentum as more mothers publicly share their stories and global public health initiatives such as Maternal Mental Health awareness campaigns increasingly use empowering and destigmatizing language (5). Today, Positive Youth Approaches (PYA) teach adolescents that they can adopt a growth mindset during their time of identity difussion. The "disorienting dilemma" of matrescence should be similarly viewed as a fertile period of self-discovery, experimentation, and subsequent mastery for mothers (55). For example, managing a central preoccupation like the desire for autonomy versus healthy interdependence can be reframed as an occasion to explore their personal values and to become more clear in their direction, meaning, and purpose. This may herald a more pivotal turning in their overall worldview, a *metanoia* that is more akin to an existential conversion-- a fundamentally transformed outlook in life, or a moral evolution.

To that end, the repackaging of the nosology outlined in **Box 1**. into an innovative self-assessment tool that acknowledges the bivalent, dual, or paradoxical nature of motherhood would help identify not only areas of vulnerability and symptoms as is the convention in a psychiatric evaluation but also the coexistence of self-perceived virtues and strengths. Unfortunately, explicitly “probing for the positive” is not how mental health practitioners are commonly trained, and as such they likely miss accounting for the subtle psychological gains (e.g., tolerance for ambiguity) experienced by mothers alongside the more obvious losses (e.g., sleeplessness). This "benign neglect" could be considered an ethical violation as the psychological and philosophical literature has validated a range of indicators for maternal subjective well-being and post-traumatic growth, and even the presence of *perinatal flourishing* (18). Denying the full range of maternal experience may have unforeseen consequences such as too many false positive diagnoses of depression in light of widespread screening. It may also unwittingly lead mothers to believe their failures are more important than their achievements. Designing a more comprehensive “positive and negative” assessment would be a useful adjunct to the widely used Whooley Screening Questions or Edinburgh Postnatal Depression Scale (EPDS). Such an innovation would also be in keeping with John Cox’s recommendation that organizations such as the Marcé Scientific Society or Global Alliance for Maternal Mental Health create forums for thinking about new scale development (56). Interestingly, in the appendix of Cox’s ten updated recommendations for the optimal administration of the EPDS are instructions on how to thoroughly ask and listen to answers:

“*When used to assess a mother in the community, the practitioner should discuss the responses with her, listen to her story, ascertain whether clinical depression or another mental disorder is present – and consider referral and/or further listening visits” (25, p.128)*.

Perhaps swapping out “another mental disorder” with “matrescence” would be a step in the right direction for a more mother-centered, supportive, and developmentally-informed attitude. An interesting challenge moving forward, therefore, is how to best build the capacity of professionals to help mothers integrate the transformative effects of matrescence into their overall self-understanding while also preparing to assist them through impaired functioning when warranted.

Box 1 **Developmental domains of matrescence.**

**Table:** 

**Conventional bio-psycho-social domains**
*Biological.* Pregnancy, childbirth, and infant feeding involve intricate hormonal cascades (e.g., progesterone, estrogen, prolactin, relaxin, cortisol, and oxytocin) that affect the brain and body much like puberty. Physical challenges such as chronic bending, lifting, feeling "touched out," and sleep deprivation can also alter body appearance and self-perception (23).
*Neurological.* Changes in the maternal brain’s structure and function indicate another phase of human neuroplasticity. Neuroscientists describe this as 'pruning and tuning,' a process that streamlines and optimizes the brain, making it more efficient, empathetic, attuned, and socio-emotionally intelligent (24). This challenges the derogatory concept of 'baby brain,' mistakenly associated with cognitive decline (25).
*Psychological.* Heightened emotional sensitivity, caretaking responsibilities, and the ‘mental load’ can overwhelm coping abilities and lead to feelings of inadequacy. Rumination, perfectionism, and hypervigilance may become maladaptive. Adjustment involves integrating identity shifts and setting more realistic expectations, leading to enhanced ego resilience over time (26).
*Social.* The arrival of children can prompt relationships to change considerably (e.g., divorce, losing/gaining friends, mending family ties) (27–31). Issues of social comparison and group belonging (e.g., social media, peer pressure, cliquish behavior) based on child-rearing preferences may also become more central and contribute to social anxieties typical of adolescence (32).
*Cultural.* Cross-cultural factors such as variations in the Value of Children (VOC) and postpartum practices, can help or hinder maternal development (33, 34). Culturally competent care respects regional preferences and helps mothers analyze influences to discover personal preferences. Such care should promote equity and inclusion across all child-rearing models and family structures, including LGBTQ+ parenting (35).
*Economic.* Mothers may face professional setbacks or unpaid labor due to their parental status, which exacerbates existing gender biases (36–38). Terms like “leaky pipeline”, “maternal wall”, or "maternal wage penalty" describe phenomena such as premature workforce exit, leading to activism for fair pay and family-leave policies (39). Others turn to “mompreneuriship” as solutions or seek roles with social impact (40).
**Expanded ideological domains**
*Moral.* Ethical concerns grow as mothers contemplate the fairness and systemic oppression within societal structures (workforce, healthcare, childcare) (41, 42). The COVID-19 pandemic further exposed these disparities and stronger advocacy for equity and justice (43). Such awakened awareness may lead to greater compassion for others, coupled with a reduced tolerance for violence in media, reflecting a deep empathy for families affected by tragedies such as war.
*Ecological.* Climate change may drive mothers to adopt environmentally conscious behaviors, ranging from recycling to engaging in political protests (44–46). They may also report an attitudinal shift, from anthropocentric to ecocentric, developing an aversion to overconsumption and a desire for a deeper connection with nature and all living beings in a reciprocal relationship (47).
*Existential*. A maternal existential perspective sees self-realization as continuously evolving, not fixed (48, 49). This reassessment of life priorities, personal freedom, and choice often connects back to the responsibilities of parenting. Establishing practical boundaries can nurture a new ethic of care that considers both mother and baby, moving from self-sacrifice to mutual interdependence (50).
*Spiritual.* An increased engagement with spiritual practices or religious faith may develop, including transformative experiences like "born again" events, or the transpersonal phenomena of indigenous traditions (e.g., synchronicities, ancestral communication). Integrating spiritual views into coping with reproductive grief (e.g., infertility, miscarriage) may offer comfort (51–54).

In conclusion, it is important to emphasize this stage of development as a critical window for establishing lifelong positive health behaviors. Developmental factors such as the timing of the first pregnancy or whether a mother has been given a proper 'head start,' by equipping her with comprehensive matrescence education and not just childbirth preparation, can profoundly impact the course of motherhood. Future maternal functioning depends in part on past exposures, with each stage affecting the next and leading to a weathering effect of cumulative distress over time. This developmental cascade requires redesigning healthcare services to include prevention and early intervention programs, seamless linkages to professionals—especially during peak times of need—and sequential, stepwise care. Such a life course perspective must also extend its focus beyond individual-level assets and consider the broader ecological systems in which mothers are situated. It should critique the quality of their own holding environment and incorporate historical factors (e.g., COVID-19). Just as with adolescence, the experiences of matrescence can be severely altered by how well or poorly a mother is socially or economically resourced, placing her on a vastly different trajectory in terms of her well-being (57).

## The promise of matrescence

Today, a growing number of scientists are studying reproductive health issues, driven by advancements in women’s representation in the sciences, as well as their increased economic and political influence. These efforts are the unfinished work of generations of scientists, many of whom were mothers themselves, who were not afforded protected time from family life or adequate funding to pursue their hypotheses in earnest. Meanwhile, matrescence has also come a long way since its initial introduction to the scientific community in the 1970s, gaining significant traction and recognition. Its resurgence has catalyzed fresh discoveries across various fields, from neuroscience to economics and has been embraced by contemporary scholars as a good fit for reframing their inquiries (58). However, this has also led to fragmentation, with numerous disconnected discussions lacking a centralized platform for a more fruitful exchange of complementary perspectives. Lewis Gordon, as cited by Mantuori, notes that

*“The emergence of disciplines has often led to the forgetting of their impetus in living human subjects” (59, p.45).*

The prospect of matrescence serving as an academic container to consolidate its disparate strands of scholarship into a newly named specialization is promising. E.O. Wilson similarly advocated for ‘a unification of knowledge … a consilience,’ since bridging the gaps between disciplines can enhance the depth and diversity of understanding of any subject (60). A parallel process to what was done for adolescence more than a century ago, now for matrescence, could promulgate the same progress (61). Cross-disciplinary collaboration is also essential for maintaining checks and balances, especially for more dominant disciplines like psychiatry that may inadvertently skew scientific discourse, social trends, or grant allocations towards its own objectives. Efforts should be *inquiry-driven* rather than *discipline-driven* as a reminder that the priority should be a common commitment to improving the well-being of mothers.

Matrescence has also captured the interest of the private sector, leading to the development of various profitable outputs such as beauty products, luxury wellness services, trade books, and expensive online educational programs. This trend has opened up avenues for its commercialization with little ethical oversight. It is therefore necessary to protect and ensure robust support for all new mothers, not just the privileged few. The evolving science of matrescence, combined with grassroots activism and a reproductive justice perspective, demands a radical reimagination of our collective obligation to empower every mother to thrive, regardless of their socioeconomic status. This will require a multi-pronged approach: 1) establishing foundational knowledge of its unique developmental tasks and coping mechanisms; (2) identifying environmental factors that either facilitate or impede its optimal progression; (3) overhauling healthcare systems to ensure greater integration of services; (4) enforcing ethical guidelines for the treatment of mothers as a protected group; 5) expanding access to public health education initiatives; 6) training competent professionals in the specialization of maternal well-being. As was once said of adolescents, until society embraces this responsibility, the promise of matrescence will remain unfulfilled for millions of women. These issues will only continue to gain importance, and with them, the need for bold ideas like matrescence that can better sustain the reproductive life satisfaction of parents and the future environmental conditions that support them.

## Data availability statement

The original contributions presented in the study are included in the article/supplementary material. Further inquiries can be directed to the corresponding author.

## Author contributions

AA: Conceptualization, Visualization, Writing – original draft, Writing – review & editing.
